# Dissecting the evolvability landscape of the CalB active site toward aromatic substrates

**DOI:** 10.1038/s41598-019-51940-0

**Published:** 2019-10-30

**Authors:** Yossef López de los Santos, Ying Lian Chew-Fajardo, Guillaume Brault, Nicolas Doucet

**Affiliations:** 1 Centre Armand-Frappier Santé Biotechnologie, Institut National de la Recherche Scientifique (INRS), Université du Québec, 531 Boulevard des Prairies, Laval, QC H7V 1B7 Canada; 2 PROTEO, the Québec Network for Research on Protein Function, Engineering, and Applications, 1045 Avenue de la Médecine, Université Laval, Quebec City, QC G1V 0A6 Canada

**Keywords:** Biocatalysis, Molecular engineering

## Abstract

A key event in the directed evolution of enzymes is the systematic use of mutagenesis and selection, a process that can give rise to mutant libraries containing millions of protein variants. To this day, the functional analysis and identification of active variants among such high numbers of mutational possibilities is not a trivial task. Here, we describe a combinatorial semi-rational approach to partly overcome this challenge and help design smaller and smarter mutant libraries. By adapting a liquid medium transesterification assay in organic solvent conditions with a combination of virtual docking, iterative saturation mutagenesis, and residue interaction network (RIN) analysis, we engineered lipase B from *P. antarctica* (CalB) to improve enzyme recognition and activity against the bulky aromatic substrates and flavoring agents methyl cinnamate and methyl salicylate. Substrate-imprinted docking was used to target active-site positions involved in enzyme-substrate and enzyme-product complexes, in addition to identifying ‘hot spots’ most likely to yield active variants. This iterative semi-rational design strategy allowed selection of CalB variants exhibiting increased activity in just two rounds of site-saturation mutagenesis. Beneficial replacements were observed by screening only 0.308% of the theoretical library size, illustrating how semi-rational approaches with targeted diversity can quickly facilitate the discovery of improved activity variants relevant to a number of biotechnological applications.

## Introduction

Successful protein engineering strategies remain strongly dependent on the availability of complementary experimental and theoretical resources. Although still a challenging task, directed evolution and structure-based semi-rational design strategies based on computational simulations have helped modulate selectivity and catalytic performance in many systems^1,2^. Additionally, new studies focusing on the relationship between structure, function and protein flexibility offer exceptional insight on the role of protein conformation in biochemical properties^3–7^. Other reliable predictors that improve our understanding of enzyme modification and mutational tolerance now include tools that combine statistical methodologies to investigate protein fitness, computational algorithms that predict beneficial or detrimental effects of mutation on protein function^8,9^, and frameworks that analyze the importance of complex biological network interactions in protein structure^10–15^. However, despite remarkable technical advancements and the development of powerful screening methodologies to explore sequence space, improvements in mutant library design and the application of sophisticated *in silico* analyses seldom integrate the advantages of both random and rational design in protein engineering strategies^16–19^.

In the present work, we outline an iterative semi-rational design (ISRD) strategy that merges structure-guided design with an empirical data-driven approach to help improve enzyme evolution. Using *Pseudozyma antarctica* lipase B as model system (CalB, previously identified as *Candida antarctica*)^20^, we combined *in silico* virtual docking and residue interaction network (RIN) analysis with experimental iterative saturation mutagenesis (ISM) to build smaller and ‘smarter’ mutant libraries, further simplifying screening efforts for the creation of protein diversity^21^. Residue ‘hot spots’ involved in enzyme-substrate complex formation were first identified by a substrate-imprinted docking procedure using bulky aromatic compounds that are poorly recognized by wild-type CalB, followed by site-saturation mutagenesis of targeted individual active-site positions^22^. After screening of individual mutant libraries for improved synthetic activity towards vinyl cinnamate and vinyl salicylate substrates, variants exhibiting improved synthetic activity were subsequently used as modeling templates to predict structural changes that favor enzyme activity improvements^23^. The iterative nature of ISRD allowed us to use active CalB mutants as biological and theoretical templates for additional rounds of design, mutagenesis, and improvement.

The CalB lipase was selected as model system because of its exceptionally robust tolerance to organic solvents and thermal stability (deactivation at 50–60 °C), making it one the most commonly employed industrial enzymes for synthetic and hydrolytic reactions in biocatalytic applications^24–28^. Its three-dimensional structure exhibits a canonical α/β hydrolase fold with a catalytic triad formed by residues S105, D187 and H224^29^. The active-site cavity is shaped as a tunnel that limits the steric positioning of bulky substrates such as triglycerides or aryl chains^30–32^. It also displays two co-localized acyl and alcohol pockets, where each subsite plays a specific role in substrate binding and recognition. As a result, the enzyme shows high specific activity and broad-spectrum affinity toward primary and secondary alcohols^33^, but significantly lower activity against bulkier substrates such as aryl, acyl or α- and β-substituted aliphatic chains^34^.

These structural features, coupled to the fact that bulkier substrates with aromatic substituents remain interesting flavor ester compounds in several biotechnological applications, make CalB a promising candidate for testing ISRD as a viable framework to efficiently modulate catalytic potential. The combined usage of library design and screening strategies presented here allowed the selection of efficient active-site remodeling variants that exhibit improved vinyl cinnamate and vinyl salicylate affinity in less than three rounds of evolution. This represents an interesting advancement to help tailor the enzyme to specific biocatalytic needs in the context of minimal resources and screening effort.

## Results

### Optimization of CalB overexpression

To develop a reliable expression system aimed at improving esterification of bulky aromatic substrates in CalB, we tested 5 *E. coli* strains in combination with a codon-optimized CalB gene expressed from IPTG-inducible T7 vector pET22b(+) (see Experimental Procedures). After standardization of strain expression, growth temperature, culture media, and IPTG concentration, the most efficient bacterial CalB producer was found to be *E. coli* Rosetta (DE3) grown on SB medium (0.1 mM IPTG induction, 16 °C) (Figs S1–S2). Using these experimental conditions, we also demonstrated that this expression system could reliably produce an active recombinant form of CalB that efficiently catalyzes esterification of 1-decanol and oleic acid (Fig. S2B), further providing an efficient solid-state screening medium for activity.

### Development of a transesterification procedure for lipase screening

Based on a previous report, we adapted and standardized a liquid medium CalB synthetic activity screening assay using vinyl analogs to mimic cinnamic and salicylic acid compound recognition^35^. Since both the cinnamic and salicylic lipase-catalyzed products of interest are devoid of distinctive spectroscopic properties relative to their respective substrates, this high-throughput screening approach exploits the release of acetaldehyde and its reactivity with 3-methyl-2-benzothialinone (MBTH) to generate the corresponding aldazine moiety (Fig. S3A). This aldazine is further converted to a blue-colored tetraaza-pentamethincyanine (TAPMC) dye under oxidative coupling with another MBTH molecule^36^. Effectively, an acetaldehyde molecule is released from each vinyl substrate analog of the lipase-catalyzed transesterification reaction, affording colorimetric detection due to derivatization with reporter compound MBTH. Absorbance measurements therefore allow proper quantification of lipase transesterification. To demonstrate the efficiency of this approach, we tested four lipase variants that mimic potential CalB mutants to be engineered in the present work, *i.e*. wild-type (WT) CalB and three previously reported variants displaying modest synthetic activity improvements against bulky substrates (S47L, L278P, and the double mutant I189A-L278P)^37–39^. Figure S3 shows that WT CalB performs better in salt-activating organic conditions and low water concentration. Due to similar synthetic performance relative to the previously reported variant CalB-L278P, we selected WT CalB as a proper starting template for all future protein engineering steps (Fig. S3D). To standardize screening conditions and to prepare the enzymatic extracts for proper organic solvent conditions, we also used 3% water content (v/v) and salt-activating conditions in potassium phosphate buffer prior to lyophilization (Fig. S3C,D).

### Iterative semi-rational evolution strategy

**Figure 1:**
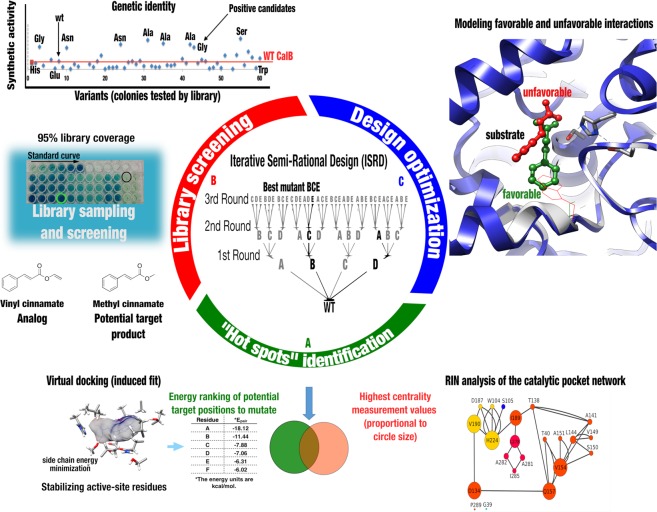
Schematic representation of the Iterative Semi-Rational Design (ISRD) strategy employed in the present work. (**A**) Identification and selection of active-site mutational ‘hot spots’ by convergence of two approaches. First, identification of residue positions that stabilize the ligand-enzyme complex is achieved by virtual docking, after which involvement of those positions in the maintenance of structure and topology of the active-site cavity is performed by a Residue Interaction Network (RIN) analysis^12^. Enzyme-substrate complex formation is accomplished by mimicking an induced fit mechanism using virtual docking. Targeted active-site positions are selected based on ranking of energy contributions for each docking complex and residue interaction likelihood with the desired substrates. RIN centrality measures provide information on residue ‘hot spots’ that are more likely to maintain CalB structure. As a result, positions with low betweenness scores are prioritized, *i.e*. positions with lower impact on protein cavity disruption. (**B**) Building and screening of the mutant libraries using a vinyl analog to measure the synthetic activity of CalB in organic solvent conditions (see Experimental Procedures for details). Genetic identification of the most active mutants allows selection of the best candidates as template(s) for the next round of mutational selection. (**C**) Modeling of experimentally selected variants with favorable active-site replacements as template for the next round of virtual docking (**A**). This complementary procedure combining experimental and computational approaches is iteratively repeated to improve esterification activity catalyzed by CalB.

To overcome limitations inherent to typical directed evolution strategies in enzyme design, we elected to use an iterative improvement framework combining a series of computational and experimental approaches to modify active-site architecture, geometry, and affinity in CalB, providing means to alter its synthetic capabilities. This iterative semi-rational design (ISRD) strategy was performed in three complementary steps (Fig. 1): (A) identification of potential ‘hot spots’ for substrate recognition and stabilization in the CalB active site using a combination of virtual docking and RIN analysis^10^; (B) experimental design and screening of mutant libraries, providing means to select variants displaying improved transesterification; (C) *in silico* analysis of the most active variant(s) by molecular modeling to provide structural and energetic feedback for the design of subsequent rounds of mutant libraries.

**Figure 2:**
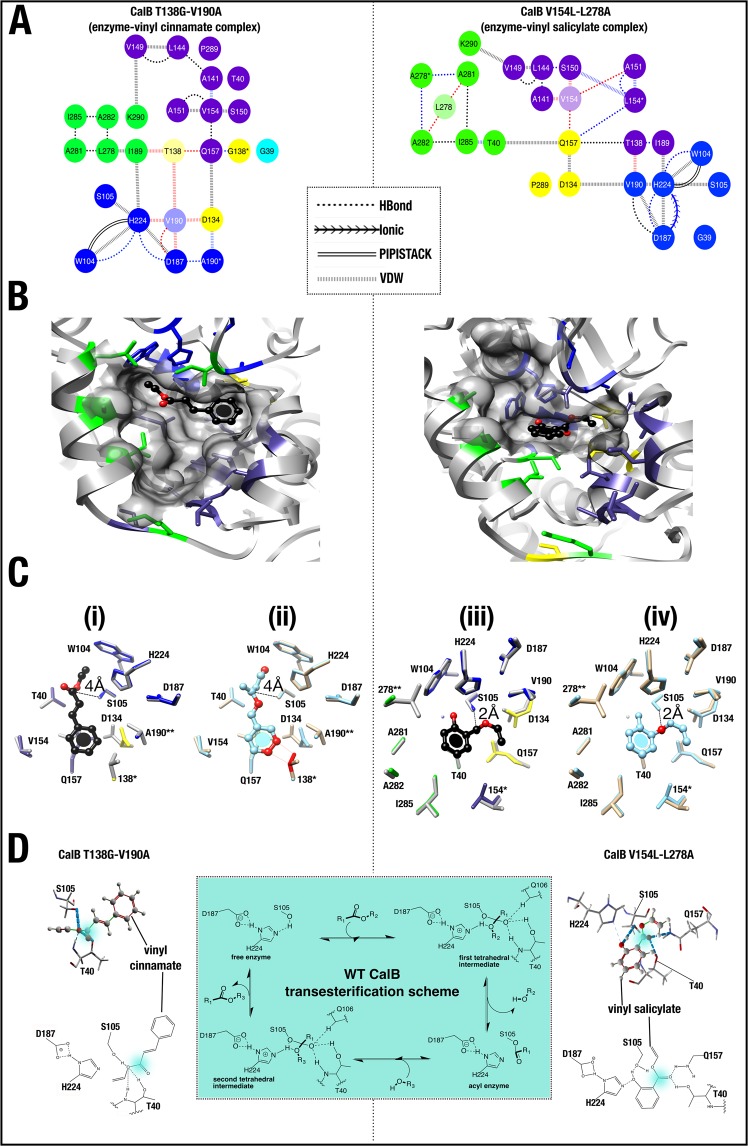
Structural changes in the active-site cavity of CalB upon mutagenesis and selection. (**A**) Changes in RINs under selective pressure shows that each evolutionary trajectory (vinyl cinnamate or vinyl salicylate) is preceded by module reorganization of the residues forming the catalytic pocket. Both pathways exhibit 4 main residue modules in the CalB cavity, identified as blue, purple, green, and yellow residue clusters sharing hydrogen bonding, electrostatic, π-π stacking, and/or van der Waals interactions. Loss (gain) of residue interactions upon mutation are labeled as red (blue) line symbols. Asterisks illustrate mutated residue positions. Each RIN illustrates the comparison between WT and variant enzyme-substrate complexes. (**B**) Modeled enzyme-substrate complexes showing how vinyl cinnamate interacts with a larger active-site cavity in mutant T138G-V190A relative to vinyl salicylate in mutant V154L-L278A. Both variants stabilize substrate molecules by exploiting distinct physicochemical properties in different active-site areas. (**C**) Both double mutants T138G-V190A and V154L-L278A favor stabilization of substrate aromatic rings, allowing proximal alignment of the carbonyl moiety 2–4 Å away from the catalytic S105 (dashed line). Sub-panels (i) and (iii) show WT side-chains in gray and mutant side-chains color-coded according to their respective module shown in panel A. Sub-panels (ii) and (iv) illustrate how active-site mutations allow for proximity with substrates by preventing atomic clashes (red dashed lines and red colored atoms). Mutant and WT enzymes are presented in blue and gray, respectively. (**D**) Formation of a transition state-like pre-intermediate between the best CalB variants and their respective substrates. Schematic view of the transesterification reaction is provided (center), with variants T138G-V190A and V154L-L278A (left and right, respectively) depicted in 3D (top) and in schematic view (bottom). H-bonding interactions are illustrated by dashed lines and the carbonyl moiety is highlighted in cyan.

Protein evolution is driven and constrained by the relationship between structure and function. For that reason, we used a combination of virtual docking and RIN analysis^12^ to guide the semi-rational design of CalB for transesterification improvement. Docking simulations were first performed to uncover which residues are directly involved in substrate positioning and stabilization prior to reaching transition state (see below). To validate how RIN analysis can reveal useful information on protein network residues that lead to advantageous substitutions, we evaluated 136 previously reported CalB mutations from a network-centric perspective (see Experimental Procedures for details). We found that more than 80% of previous beneficial mutations that either increase CalB activity or improve protein stability exhibit low values of betweenness centrality measure^12^. Since centrality measure identifies which residues exert more control over the RIN, we identified which amino acid replacements stabilize the enzyme-substrate complex and have lower chances of disrupting the RIN structure upon mutation. In other words, amino acid replacements exhibiting high energy contributions to binary complex stabilization and low values of betweenness centrality measure were selected as promising positional targets for mutagenesis. To challenge this approach, we have also tested a negative control exhibiting high energy contributions and high betweenness centrality measure (Fig. S5). Since the MolDock scoring function and the substrate-imprinted docking procedure both consider conformational sampling of residue side-chains involved in the docking complex, only the theoretically active configuration of the catalytic triad (D187, H224 and S105) was fixed during docking, thus mimicking transition state orientation^40^. To differentiate the importance of individual residue contributions, the energy cut-off was set to −1.0 kcal/mol (Table 1). Our RIN analysis shows that the catalytic cavity of CalB is divided into three main residue modules, which describe clustering interactions defined by the Linkland algorithm^41,42^ (Fig. 2). Systematic mutational exploration of these CalB active-site modules was performed over the course of all three generations presented in this work (Fig. S6).

**Table 1:** Binding energy contributions of enzyme-substrate complexes for WT CalB and the most efficient CalB variants selected after three rounds of ISRD.

Ranking	Vinyl cinnamate				Vinyl salicylate			
	WT CalB		T138G/V190A		WT CalB		V154L/L278A	
	aa	kcal/mol	aa	kcal/mol	aa	kcal/mol	aa	kcal/mol
**1**	T40	−18.31	T40	−17.49	T40	−16.96	T40	−19.70
**2**	Q157	−14.85	Q157	−15.82	Q157	−11.18	I189	−16
**3**	I189	−8.71	I189	−11.31	S105	−9.76	H224	−9.79
**4**	S105	−6.77	H224	−6.81	I189	−8.92	Q157	−9.66
**5**	H224	−6.73	S105	−5.24	H224	−7.71	D134	−5.18
**6**	V154	−5.52	D134	−4.13	D134	−5.51	V190	−4
**7**	T138	−5.08	G138*	−3.61	W104	−3.61	W104	−3.6
**8**	D134	−4.36	G39	−3.45	T138	−3.37	I285	−2.28
**9**	G39	−3.65	W104	−2.85	V154	−3.05	A281	−2.24
**10**	W104	−3.60	V154	−2.62	V190	−2.99	G39	−2.2
**11**	A141	−2.22	A190*	−2.32	G39	−2.95	L154*	−2.05
**12**	V190	−1.39	A141	−1.9	I285	−0.90	T138	−1.76
**13**	I285	−1.22	I285	−1.01	Q106	−0.6	Q106	−1.48
**14**	T158	−1.04	T158	−0.87	A141	−0.41	G41	−0.46
**15**	G137	−1.02	A281	−0.79	A281	−0.38		
**16**	A281	−0.93	L278	−0.66				
**17**	L278	−0.81	Q106	−0.3				
**18**	T42	−0.54						
	S153	−036						
**Total energy of enzyme-substrate complexes**	−**82.74 kcal/mol**		−**83.27 kcal/mol**		−**67.15 kcal/mol**		−**76.31 kcal/mol**	

Note: Asterisks specify mutations that were selected for additional rounds.

Virtual docking of both cinnamic and salicylic acid analogs generated a number of energetically favorable interactions between the enzyme and substrates, highlighting individual ranking energy contributions for each residue position (Table 1). For the first generation of mutants (G1), we designed 6 distinct site-saturation mutagenesis libraries at CalB positions 40, 134, 138, 154, 157, and 189, which were then individually screened against both substrate analogs. These semi-randomized positions were selected according to favorable energy contributions with respect to enzyme-substrate complex stabilization, increasing the likelihood of obtaining positive variants^43^. Residues H224 and S105 were excluded from randomization because of their functional importance in the catalytic triad. Since ISRD combines empirical results with a predictive modeling phase where mutations that increase activity also alter enzyme cavity size and shape, each new design round represents a new template of virtual docking (Fig. 1A). Indeed, enzyme-substrate complexes can alter existing RIN connections or gain new interactions that stabilize ligands in the active-site cavity. Consequently, the use of different templates at each round of protein design can expand the original search space. As a result, mutational exploration of the active-site cavity was expanded to CalB positions 39, 104, 190, 278, and 285 for subsequent library generations (G2 and G3). For G1 and G2, single-site saturation libraries were individually screened against each substrate analog. For G3, we reduced the number of positions explored to investigate 3 individual single-site saturation libraries for two distinct templates obtained from previous generations. G3 also explored the combination of successful mutations that were independently selected in G1 and G2.

### CalB library screening

**Figure 3:**
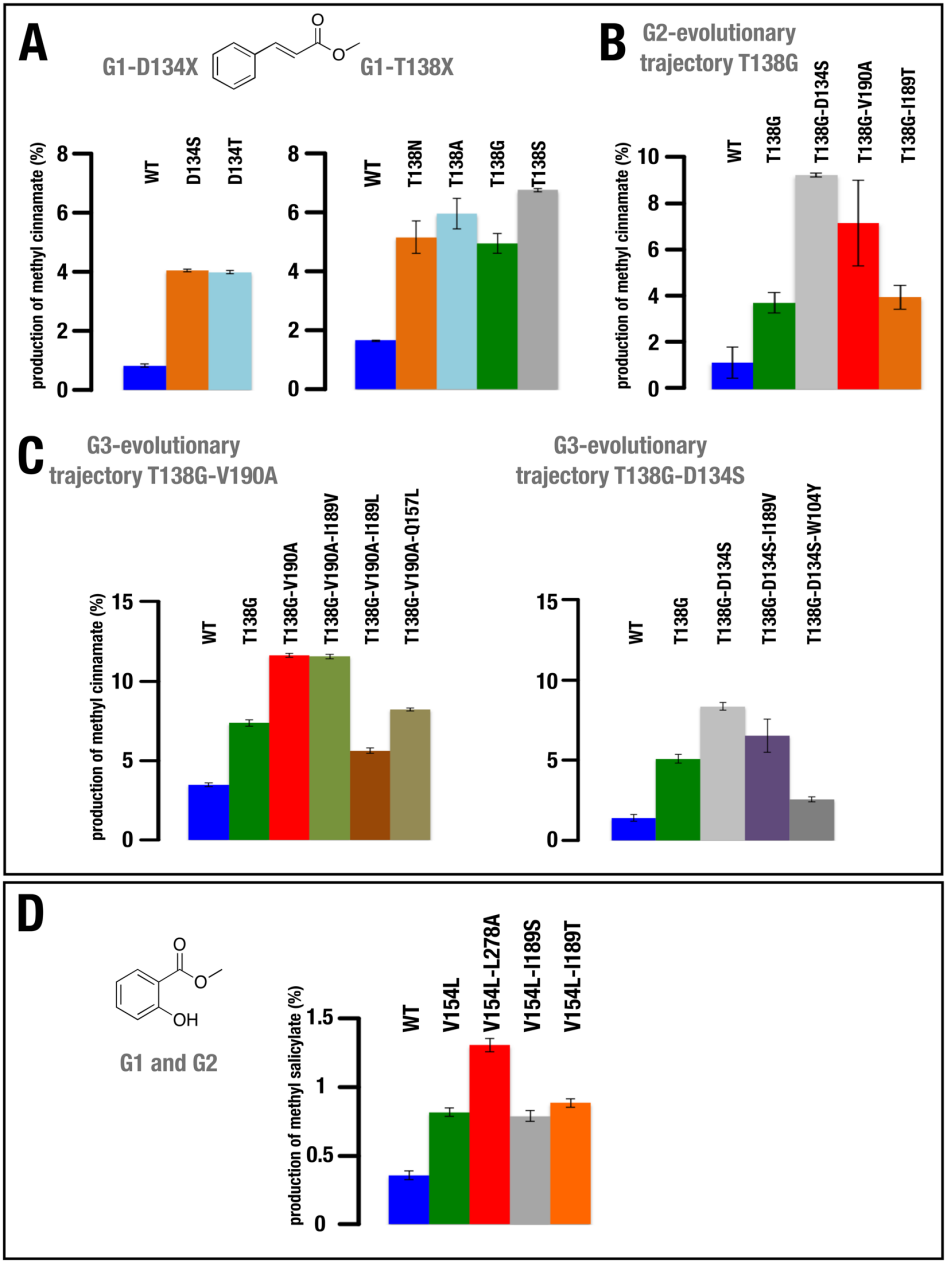
Methyl cinnamate and methyl salicylate production by CalB lipase subjected to 3 rounds of Iterative Semi-Rational Design (ISRD). (**A**) First-generation (G1) selection of 6 improved variants towards methyl cinnamate production at positions 134 and 138. (**B**) Additive effects of second-generation (G2) mutations at positions D134S, V190A, and I189T over the G1-selected T138G template. (**C**) Exploration of two evolutionary trajectories from starting templates T138G-V190A and T138G-D134S as part of the third generation (G3). (**D**) Summary of the best variants selected from 2 CalB ISRD generations (G1-G2) performed with vinyl salicylate as substrate. Each experiment was performed in triplicate.

Our screening efforts on G1, G2, and G3 libraries illustrate that the evolutionary trajectories of both substrates show significant differences with respect to favorable mutational solutions that benefit methyl cinnamate and methyl salicylate transesterification (Fig. 3). In accordance with our computational energy calculations (Table 1), library design for the methyl cinnamate trajectory was more successful at predicting positive variants, generating 14 CalB variants with increased synthetic activity relative to WT CalB (Fig. 3A–C). In contrast, the methyl salicylate trajectory generated 3 variants with increased activity relative to WT CalB (Fig. 3D). Interestingly, the most active methyl cinnamate G1 variants were observed for randomized positions T138 and D134 (Fig. 3A), where 6 variants showed increased transesterification activity relative to WT. The most efficient G1 variants are T138G, T138A, T138S, and T138N, displaying up to 4-fold increase in methyl cinnamate production relative to WT CalB (Table SI). The addition of G2-selected mutations (V190A and D134S) to the G1-T138G template allowed selection of double mutants displaying production yields nearly 6-fold that of the WT conversion rate for the same compound (Fig. 3B).

In contrast, the salicylate evolutionary trajectory yielded mutants with lower synthetic activity enhancements (Fig. 3D). With only 2 designed library generations, we nevertheless selected a double mutant (V154L-L278A) exhibiting 3.6-fold increase in catalytic activity relative to WT CalB. Noteworthy is the fact that WT CalB shows negligible synthetic activity against vinyl salicylate (<0.5% conversion). These modest enhancements thus remain promising avenues for additional rounds of design, allowing selection of active variants with respectable activities for desired substrates. We found similar mutational patterns with both substrates, whereby 2 rounds of ISRD with 2 single replacements increased transesterification against aromatic substrates unrecognized by WT CalB.

For G3 library design, we opted to combine positive mutations selected in G1 and G2 to investigate additive or synergistic behavior on catalytic activity. As a result, two initial G1-G2 templates were used for further active-site exploration: T138G-V190A and T138G-D134S (Fig. 3C). Contrary to expectations, combining the two most active G2 substitutions (D134S and V190A) in the context of the favorable G1 single mutation T138G had deleterious effects on the performance of CalB towards methyl cinnamate synthesis (Fig. S4A). The lack of a synergistic effect after combination of the two beneficial mutations V190A and D134S could potentially be explained by changes in the RIN, influencing propagation of long-range protein dynamics and/or local residue packing, further indirectly affecting enzyme activity. Our RIN analysis of the active-site cavity in CalB showed that the local network of interactions is overwhelmingly unaffected by the introduction of the D134S substitution in the context of double variant T138G-V190A (Fig. S4B). However, the effects of such substitution propagate outside the active-site cavity, perturbing the core of the protein network (Fig. S4C). More precisely, D134S reorganizes the core network and alters a set of 10 residue interactions (Fig. S4C, green lines).

**Figure 4:**
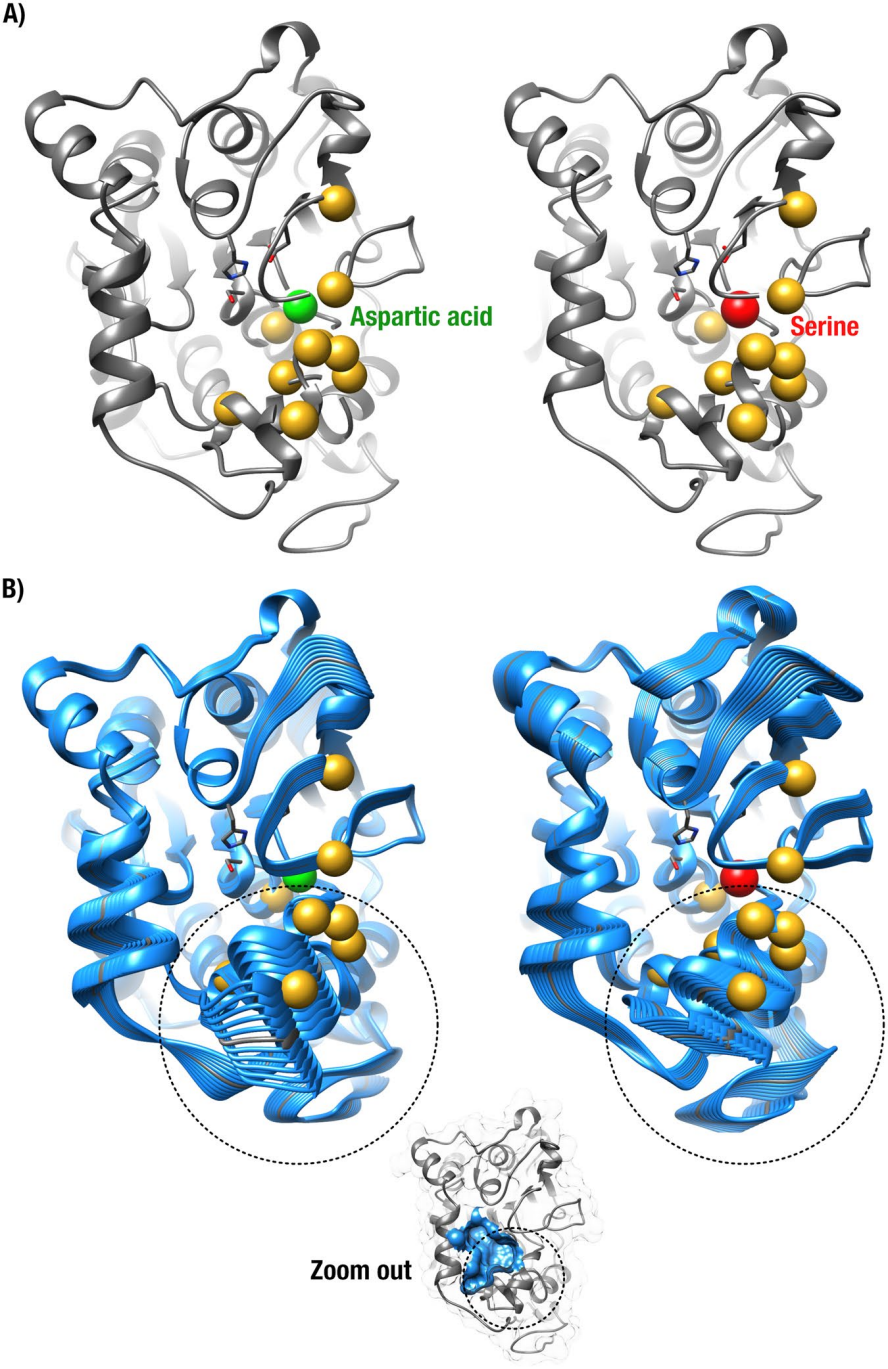
Comparison of RIN and Normal Mode Analysis (NMA) for CalB (T138G/V190A) and CalB (T138G/V190A/D134S). (**A**) Ribbon diagram comparison between double mutant T138G/V190A before (left) and triple mutant T138G/V190A/D134S (right). D134 and S134 are shown as green and red spheres, respectively. Residues affected by the loss of RIN connections after introduction of the D134S substitution are shown as gold spheres (green connections in Fig. S4C). Side-chains of the catalytic triad are shown as sticks. (**B**) Trajectories obtained from Normal Mode Analysis (NMA) of double mutant T138G/V190A (left) and triple mutant T138G/V190A/D134S (right). The main differences in large-scale conformational exchange between both variants is concentrated in the structural motif marked by a black circle. Relative location of this motif relative to the active-site cavity is illustrated by the small structural bottom inset (active site in blue surface representation).

Comparisons between the two energy-minimized structures of double mutant T138G-V190A and triple mutant T138G-V190A-D134S illustrate overall fold conservation and minimal structural perturbation in CalB (Fig. 4A). However, Normal Mode Analysis (NMA) of both double and triple variants suggests that the D134S substitution can initiate large-scale rigidification of the functional motions experienced by CalB (Fig. 4B). Indeed, our NMA results illustrate significant conformational exchange alterations for a set of 10 residues that co-localize with many residues involved in the interaction network (Fig. S4), in addition to inducing large-scale conformational changes in residues of the active-site cavity (Fig. 4B). This observation could partly explain the deleterious effects introduced by the D134S substitution in the context of otherwise beneficial mutations T138G-V190A. Similarly, amino acid replacements I189V, I189L, and Q157L were the only G3 substitutions providing increased synthetic activity relative to WT (Fig. 3C), while any other substitution in the T138G-V190A evolutionary trajectory decreased activity below WT levels. Because our G3 screening effort was reduced to only 3 libraries for 2 CalB templates (T138G-V190A and T138G-D134S), none of the G3 variants outperformed the best G2 candidates.

### Natural vs. directed evolution of the CalB active site

**Figure 5:**
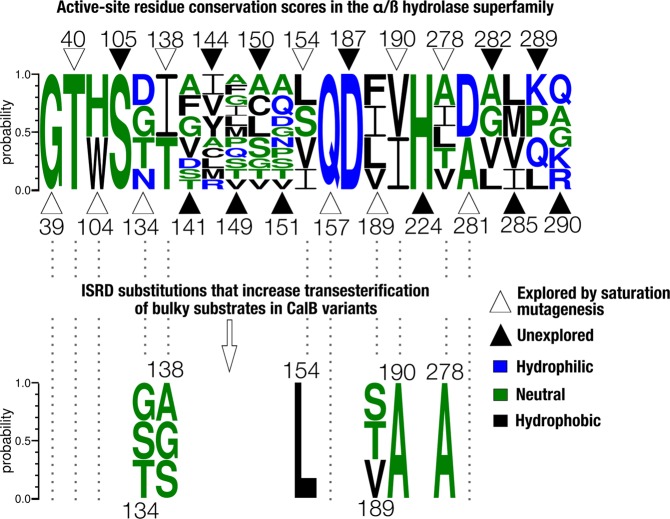
Comparative analysis of α/ß hydrolase natural sequence diversity relative to ISRD-selected active-site mutations that increase CalB transesterification on bulky substrates. Relative frequency conservation scores per position are presented for active-site residues across the α/ß hydrolase superfamily. Open triangles represent active-site residues subjected to saturation mutagenesis in the present work, while closed triangles represent unexplored active-site residues. Hydrophilic, neutral, and hydrophobic residues are colored blue, green, and black, respectively. Figure was prepared with the WebLogo server using CalB sequence numbering^74^.

We randomly explored 11 positions out of the 23 residues forming the active-site cavity of CalB (Fig. 5). Despite the low water content and hydrophobic nature of the transesterification reaction performed under organic solvent conditions, the most favorable residue replacements selected in our screening efforts did not translate into strict hydrophobic requirements. For both substrates, serine and alanine were the most common replacements selected after 3 rounds of ISRD. As expected, smaller amino acid replacements were found in the most active variants, a mandatory requirement to accommodate bulkier substrates in the active-site cavity of CalB. An evolutionary trace analysis (ETA) revealed that either serine at positions 134, 138 and 189 or alanine at position 190 represented two new unexplored solutions in the natural diversity of the α/ß hydrolase superfamily (Fig. 5). Consistent with the conservation score obtained for residues 39, 40 and 157, no replacements at these positions favored synthetic activity or tolerated significant sequence diversity. These findings are also consistent with our binding predictions for the vinyl cinnamate and vinyl salicylate analogs, whereby T40 could potentially act as the stabilizing oxyanion hole over the course of the catalytic reaction with both substrates (Fig. 2D). These results also support the fact that T40 is the most important energy contributor during formation of the enzyme-substrate complex (Table 1). However, selective pressure to preserve glycine at position 39 might be related to a smaller side-chain requirement rather than direct contribution to substrate stabilization in the active-site cavity (~3.0 kcal/mol, Table 1).

## Discussion

The lack of a proper lipase selection system or any reliable high-throughput screening methodology to quantify transesterification improvements of desired substrates inspired us to develop an alternative approach to explore the mutational landscape of CalB. This ISRD approach employs vinyl substrate analogs of the highly-sought flavoring agents methyl cinnamate and methyl salicylate, in addition to concentrating mutations in the active-site cavity, *i.e*. where they are most likely to be effective^43^. As a result, ISRD focuses on improving the design of smaller and ‘smarter’ mutant libraries, rather than blindly increasing screening capabilities. As proof of concept, we tested this approach using the CalB lipase against bulky aromatic substrates vinyl cinnamate and vinyl salicylate. This was justified by the fact that WT CalB natively recognizes long aliphatic substrates with low commercial appeal, while exhibiting poor synthetic activity on bulkier, branched, or aromatic substrates of significant biotechnological interest like the methyl cinnamate and methyl salicylate products synthetized in this work. Additionally, unlike hydrolysis, transesterification reactions are typically performed in harsh organic solvent conditions that further mimic industrial production, which often require stronger tolerance to enzyme denaturation.

In the present work, exploration of 11 out of the 23 active-site cavity positions in CalB was performed, uncovering improved single, double, and triple mutants from the combination of individually saturated positions. Considering that the total sequence space exploration would have generated 116,840 enzyme variants (23 positions to explore for single, double, and triple variants), testing 360 CalB variants per evolutionary trajectory (6 libraries per generation, *i.e*. 120 CalB variants for 3 generations) only represents a small fraction (0.308%) of the total putative effort required to screen the entire sequence space^44^. Nevertheless, screening of an experimentally modest 60–66 bacterial transformants per library over 3 generations yielded 12 mutants exhibiting improved CalB transesterification relative to WT (Figs 3 and 4). These results illustrate that the ISRD approach uncovered newly productive CalB variants, acting as an efficient method to quickly engineer enzyme properties with minimal screening effort. ISRD also advantageously compares to the 6-fold hydrolytic improvements obtained from massive screening efforts incorporating microfluidic water-in-oil droplet compartment platforms that sort 3 × 10^7^ variants per round of directed evolution^45^.

ISRD also allowed CalB synthetic improvements towards bulkier substrates. In that regard, the approach outperformed results obtained in other CalB engineering strategies, whereby screening efforts often exceeded 1,000 explored mutants to select ~10–32 improved variants^32,46,47^. Because ISRD combines virtual docking with ISM, it also considerably helped reduce total screening effort. This advantage even holds true in comparison with approaches that solely rely on *in silico* screening methodologies. For instance, Juhl and coworkers used a library of 2,400 CalB variants to generate 11 improved CalB mutants^31^. Similarly, the closest screening effort comparison with the present work was provided by a study carried out by Wu and coworkers^34^, whereby an ISM mutagenesis protocol was performed on a library size of 430 colonies per generation.

The present work also allowed identification of previously unknown synergistic, antagonistic and compensatory mutations in the active-site of CalB. These include synergistic substitutions like the G1 mutations T138G and D134S observed in the methyl cinnamate evolutionary pathway (Fig. 3A,B), in addition to the antagonist relationship between independently favorable mutations like the adverse combination of V190A and D134S (Figs 4 and S4). They also include the compensatory effects of double mutant T138G-V190A on substitutions at positions 189 and 157 (see triple mutants with I189L or Q157L in Fig. 3C, all of them explored with negative effects on the synthetic activity of CalB in G1). Results for both substrate trajectories suggest that one of the main challenges in CalB design is shaping cavity size to accommodate bulkier, aromatic substrates. Indeed, redesign of the active-site cavity appears critical for proper positioning and orientation of the substrates to achieve transition-state stabilization during catalysis. For the cinnamate trajectory, size reduction of specific side chains (Thr and Val replaced by Gly and Ala, respectively) provided sufficient room for proper catalysis (Fig. 2C,D). Our modeling work suggests that this could be achieved by rearrangement of the surface cavity in the vicinity of positions 134, 138 and 190, resulting in an insignificant increase in energy of the enzyme-substrate complex (Table 1). This observation suggests that synthetic activity improvements rely on proper substrate accommodation in the active-site cavity rather than important increases in enzyme-substrate affinity. Interestingly, vinyl salicylate activity correlates with a decrease in active-site cavity volume (Table SII). This reshaping still provides proper accommodation of the substrate, translating into increased theoretical stabilization of the enzyme-substrate complex energy (Table 1).

The distribution of evolutionarily conserved residues among homologous folds of the lipase and α/ß hydrolase superfamily allowed comparison of our results with solutions exploited by nature under different selective pressure environments (Fig. 5). Although such evolutionary information can be carefully considered prior to performing directed evolution, natural diversity rarely represents the best solution in a different evolutionary context. Indeed, our results confirm that favorable substitutions can be introduced at otherwise highly conserved positions subjected to different selective pressures. This is illustrated by mutations at positions 138 and 190, both highly conserved across the α/ß hydrolase superfamily (Fig. 5). To the best of our knowledge, no other studies have reported beneficial effects on the synthetic activity of CalB for replacements D134S, D134T and T138G. Similarly, although D134N, T138A, T138N and T138S mutants have been shown to promote catalytic efficiency against longer chain substrates, characterization of their synthetic performance was never reported^31^. On the other hand, although mutations T40A and T40V were suggested as potentially important enantioselective contributors, we did not observe these substitutions in any of our evolutionary pathways, probably due to the importance of T40 as an essential enzyme-substrate complex contributor^48,49^. Similarly, single substitutions Q157A, I189V and I189A were also previously reported as replacements that increase catalytic activity and reduce potential steric clashes with bulky substrates. In our screening conditions, I189V was the only replacement tolerated, although it was ineffective at increasing synthetic activity in the context of a triple mutant including T138G and V190A (Fig. 3C)^50–52^.

To the best of our knowledge, the present work represents the first report incorporating a RIN analysis in the context of a protein engineering framework. This analysis revealed itself useful to investigate amino acid coordinates resulting from mutation in the catalytic pocket of CalB. Our RIN analysis suggests that two additional hydrogen bonding interactions may help stabilize enzyme-substrate complex formation in both evolutionary trajectories (T138G-V190A and V154L-L278A). These would include the participation of W104 and catalytic triad residues D187 and H224 (Fig. 2A, blue modules). While H224 is directly involved with molecular coordination of the catalytic serine and target atom during transesterification^32,53,54^, replacement at position W104 leads to drastic reduction of the beneficial effects conferred by mutations T138G-D134S (Fig. 3C). Previous reports have indicated that W104 could be involved in the formation of a small substrate-binding pocket, in stereoselectivity selection of secondary alcohols, while its replacement by histidine causes an important reduction of CalB activity^32,55,56^. The importance of this tryptophan at position 104 also correlates with a high degree of conservation observed at this position across the α/ß hydrolase superfamily (Fig. 5). Our results also suggest that W104 plays a determinant role for coordination and orientation of catalytic triad residues, namely through formation of new H-bonding interactions with H224, but also for substrate stabilization in both complexes (see energy contributions of complex formation in Table 1).

Prior work performed on other substrates in analogous evolutionary conditions have illustrated the difficulty of engineering modest CalB transesterification improvements^57–61^. Results highlighted here illustrate the benefits and feasibility of using ISRD as an efficient semi-rational approach to improve CalB transesterification of non-natural compounds exhibiting promising flavor ester applicability. For both evolutionary trajectories (and particularly for methyl cinnamate), synthetic improvements were achieved after a single round of semi-rational evolution, exhibiting competitive enzymatic enhancements relative to similar reports using commercial lipase sources. In the context of a production workflow involving unpurified cellular extracts as a cheap and easily scalable source of enzyme production, coupled to realistic transesterification conditions using co-solvent mixtures of methanol and *tert*-butanol, the present approach illustrates how CalB can be evolved for efficient and environmentally friendly biotechnological applications.

## Experimental Procedures

### DNA constructs and expression systems

A codon-optimized gene of wild-type CalB was synthesized for optimal expression in *E. coli* (GenScript) and cloned into expression vector pET22b+ (MilliporeSigma). A C-terminal 6-histidine Tag was designed to facilitate the purification process of all CalB variants. Using the same codon-optimized WT CalB sequence, three previously reported mutants of CalB displaying increased activity to bulky substrates in nonaqueous conditions were also synthesized: S47L, I189A, and double mutant I189A-L278P^38,39,46^. Overexpression of the CalB enzyme from IPTG-inducible vector pET22b+ was tested using 5 different *E. coli* strains: Rosetta-Gami (DE3), Rosetta (DE3), Rosetta 2 (DE3) PLacI, Origami 2 (DE3), and BL21 (DE3) (MilliporeSigma). The selection markers employed were a combination of kanamycin and chloramphenicol (Rosetta-Gami (DE3)), chloramphenicol (Rosetta (DE3) and Rosetta 2 ((DE3)-PLacI), and streptomycin (Origami 2 (DE3)). Carbenicillin was used as resistance marker for expression vector pET22b+, which was electro-transformed into each strain using 0.1-cm Gene Pulser/MicroPulser electroporation cuvettes (Bio-Rad) and an ECM 630 electroporator (BTX Harvard Apparatus). Cells were recovered in SOB medium^62^ supplemented with 20 µL of 2 M glucose (filter-sterilized) and 2 M magnesium (1 M MgSO_4_ and 1 M MgCl_2_, autoclave-sterilized).

### WT and mutant CalB overexpression

All CalB constructs were initially pre-cultured overnight in Luria Bertani (LB) medium at 37 °C and 250 rpm^62^. Expression cultures of 5 ml were then performed in two different liquid growth media (LB and Super Broth, or SB) incubated at 16 °C and 37 °C for 16 hours (250 rpm). Protein expression was induced at *A*_600nm_ = 0.5–0.6 upon addition of varying concentrations of IPTG (0.1 to 1 mM). SB medium was prepared using 32 g of polypeptone (Bio Basic), 20 g yeast extract (ThermoFisher), 5 g NaCl (Bio Basic), and 5 ml 1 M NaOH in a total of 1 L of water^37^. After incubation, cells were collected by centrifugation (21,000 *g*, 5 minutes, 4 °C) and resuspended in 200 µL of denaturing buffer for sonication (750 µL of double-distilled water, 200 µL of 10% SDS, and 50 µL of 1M DTT). After sonication, the culture supernatant was separated by centrifugation (21,000 *g*, 20 minutes, 4 °C) and cell pellets were resuspended in 200 µL of the same denaturing buffer. All protein supernatant and pellet fractions were quantified and analyzed using SDS-PAGE (Mini-PROTEAN TGX Precast Gels, Bio-Rad) and Western-Blot analyses using an anti-His-Tag HRP conjugate mouse monoclonal antibody (Qiagen) and a TMB substrate (G-Biosciences).

### WT and mutant CalB esterification assays

To confirm proper refolding and enzymatic activity of WT CalB and mutant forms, esterification assays were performed using a preculture of *E. coli* Rosetta (DE3) incubated overnight at 37 °C. Cells from the preculture were diluted and inoculated to obtain individual colonies on an LB agar plate containing carbenicillin as resistance marker and a combination of oleic acid and 1-decanol (Sigma) as substrates for the enzymatic reaction to occur (15.62 g of each substrate per liter of medium). A concentration of 0.001% (w/v) rhodamine B (R-6626 rhodamine O) was used as reporter of esterification activity, as previously reported^63^. Agar plate incubation was performed for 72 hours at 30 °C.

### Enzyme lyophilization

Selected colonies were picked and grown overnight in liquid LB medium at 37 °C (250 rpm) with carbenicillin as resistance marker. Precultures were used to inoculate a final volume of 5 ml SB culture in presence of carbenicillin, using the overexpression procedure described above (0.1 mM IPTG induction, 48 h incubation, 16 °C, 250 rpm). After incubation, cells were centrifuged at 2,400 *g* (4 °C, 15 minutes) and then resuspended in phosphate buffer (50 mg K_2_HPO_4_, 4.9 g KCl dissolved in 200 ml MilliQ H_2_O to reach a final concentration of 98% w/w)^64^. Buffer pH was adjusted to pH 7.0 using 100 mM HCl. Samples were sonicated on ice for 15 seconds and centrifuged at 2,400 *g* (4 °C, 20 minutes) to extract cell supernatant. Lyophilization of the supernatant was performed for 72 hours at −56 °C and the final lyophilized samples were stored at 4 °C in a desiccator with drierite for future enzymatic assays.

### Virtual docking of substrates in the CalB active site

To identify which amino acid residues interact with the substrate in the active-site binding cleft of CalB, a substrate-imprinted docking approach was employed^22^. This approach is based on the simulation of an induced fit mechanism between the enzyme and its substrates upon ligand binding. The process involves 2 rounds of virtual docking optimization, where the most energetically favorable structure of the first round is used as a template for side-chain energy minimization in the catalytic pocket of the enzyme. Calculations employed the Piecewise Linear Potentials for steric and hydrogen bonding interactions, and the Coulomb potentials for electrostatic forces^40^. The Nelder-Mead simplex iteration algorithm was used for all steps of energy minimization. This algorithm independently minimizes a specified number of steps for each residue, and then performs a global energy minimization of all residues simultaneously. The second round of virtual docking was performed using the newly calculated configuration of the CalB active-site binding pocket. The 5 most energetically favorable active-site configurations from the second round of virtual docking were sampled and compared to obtain the best residue targets guiding the site-directed saturation mutagenesis and experimental library design. The MolDock docking scoring function was used for all virtual docking simulations. Ligands methyl cinnamate (PubChem CID 637520) and methyl salicylate (zinc_490 mol file) were used against the crystallographically-resolved CalB template (PDB entry 1TCA)^29^. The scoring function is based on the guided Differential Evolution (DE) hybrid search algorithm, in combination with a cavity prediction algorithm^40^. All computational work was realized with the Molegro Virtual Docker 6.0 suite without incorporation of water molecules. To maintain search robustness, 20 rounds of iterations were performed for each docking procedure.

### Library Design Strategy

Based on virtual docking predictions, an individual saturation mutagenesis library was designed for each selected position in the active site of CalB. For every position identified, a specific saturation mutagenesis library derived from the WT CalB sequence was acquired by gene synthesis. Libraries for the first two generation screenings of both substrates (cinnamic and salicylic substrate analogs) were obtained through the gene synthesis service of Life Technologies (Thermo Fisher Scientific). The third generation library for both evolutionary pathways was obtained by the Gibson method^65^, in combination with a combinatorial oligonucleotide method using degenerate codons NDT, VHG and TGG. A codon bias reduced the number of necessary codons from 64 to 22, thus balancing the proportion of mutations and avoiding over-representation of certain amino acids within the libraries^66^. This strategy was coherent with the Iterative Saturation Mutagenesis (ISM) approach (Fig. 1, center), where the best mutations selected in one round are fixed and used as starting template for subsequent library design. This approach aims to reduce screening efforts and maximize the selection of beneficial mutations^21^.

### Screening of mutant libraries and transesterification assays

Screening efforts required to statistically explore the diversity of the different designer libraries and was estimated using the following equation:

$$F=1{-e}^{-{\rm{L}}/{\rm{V}}}$$

where F is the fractional completeness, V the number of possible sequence variants, and L the number of clones required to reach fractional completeness^44^. We used the ISM strategy, whereby independent single positions are mutated to saturation^21^. Using the degenerate codon strategy explained above, 22 possible codon combinations (V) were obtained, with a 95% fractional completeness (F). Consequently, our screening effort required sequencing of 66 clones per library to explore 95% diversity:

$$\begin{array}{c}F=0.95\\ \Downarrow \\ F=1{-e}^{-{\rm{L}}/{\rm{V}}}\\ \Downarrow \\ 0.95=1{-e}^{-{\rm{L}}/22}\\ \Downarrow \\ {\rm{L}}=(22)\,\ast \,\mathrm{Ln}(0.05)\\ \Downarrow \\ {\rm{L}}=(22)\,\ast \,(2.99)\\ \Downarrow \\ {\rm{L}}=65.78\end{array}$$

Sixty-six colonies were randomly picked and sequenced in each library, thus ensuring 95% coverage where each mutant is represented at least once^44^. Primers T7 (5-TAATACGACTCACTATAGGG-3) and T7terminator (5-GCTAGTTATTGCTCAGCGG-3) were used to verify the genetic identity of all CalB variants. DNA sequencing of 20 independent colonies was used to confirm sequence coverage of each library. Each colony picked (*i.e*. each CalB variant) was expressed as described above. After lyophilization, enzymatic screening was performed on cell extracts (5 mg per sample) using 2-methyl-2-propanol (*tert*-butanol) (Sigma) as organic co-solvent. Substrates were used on a 3:1 ratio of methanol (Sigma) and vinyl cinnamate (or vinyl salicylate) (Aldrich), two analogs of the target substrates cinnamic acid and salicylic acid. Enzyme assay conditions were fixed at 100 mM vinyl cinnamate (or vinyl salicylate) and 300 mM methanol. Samples were incubated at 30 °C for 2 hours and 1,000 rpm agitation in a final volume of 0.5 ml. Deionized water content was fixed at 3% v/v. After reaction completion, 3-methyl-2-benzothianoline (MBTH) was used as reporter to detect the stoichiometric ratio (1:1) between released acetaldehyde and the reduction of one molecule of vinyl analog^35^. Enzymatic samples were first diluted in deionized water (between 20 and 250 times). 200 µl of the diluted solution was further mixed with 200 µl MBTH solution (0.1% m/v in deionized water) and incubated for 10 minutes at 30 °C and 200 rpm. After incubation, 80 µl of the derivatization solution was added (1% m/v H_4_FeNO_4_S_2_*H_2_O in a solution of 0.1 M HCl prepared with deionized water) and incubated for an additional 30 minutes at 30 °C (200 rpm). Acetaldehyde concentration was obtained by monitoring absorbance at 600 nm with a Tecan Infinite M1000 PRO microplate reader. A standard curve was calibrated using 0.05, 0.1, 0.2, 0.3, 0.4, and 0.5 mM acetaldehyde.

### Molecular visualization and analyses

UCSF Chimera (version 1.1) was used for visualization and molecular analysis of all structures^7^. The RosettaBackrub server was used to predict mutational effects on the protein backbone, surface, and volume of the catalytic pocket in CalB after site-saturation mutagenesis^23^. The CASTp service was employed to analyze topographic surface changes in the catalytic cavity upon mutation^67^.

### Residue interaction network (RIN) analysis

The Evolutionary Trace Analysis was used to obtain conservation scores for residues of the α/ß hydrolase superfamily with the ConSurf server^68^. Residue Interaction Network analysis was performed with the Cytoscape 3.5.1 suite^69^ and the use of applications RINalizer^10^, Moduland2^42^, StructureViz^70^, and SetsAPP^71^. Energy minimization for all templates was performed using the YASARA force field^72^. Residue networks were calculated with the Residue Interaction Network Generator of the RING server^73^. Hydrogen bonding interactions, Van der Waals (VDW) forces, disulphide bridge formation, salt bridges, π-π stacking, and π-cation energies were considered to estimate residue connecting edges (network nodes). Bond angles and energies were calculated using the default settings of the server. The WebLogo server was employed to generate sequence images of the evolutionary conserved residues (Fig. 5) from the ConSurf server and the best mutations selected in this project^74^. To validate how RIN analysis can reveal useful information on protein network residues that lead to advantageous substitutions, we retrieved the entire published records of 136 CalB mutations of the existing literature from the 3DM database^75^. We used that information to correlate beneficial mutations (over protein activity and stability) and their centrality measures, obtained from the residue interaction network of CalB. For clarity, *degree centrality* was calculated for every node (residue) in the protein network. This was determined by the number of connections (physical interactions) of this node with adjacent neighbors (*i.e*. more physical interactions correlate to higher degree centrality). To calculate betweenness values, which identify which residues exert more control over the network, we subjected the system to an algorithm that calculates the path with the shortest distance interconnecting two nodes within the network, also known as the *geodesic distance*^10^. Another way to understand this is to investigate how many times certain nodes can perturb the shortest path between a pair of nodes within the CalB residue network (*i.e*. the more a node can interrupt a shortest path, the higher its betweenness value).

### Normal mode analysis (NMA)

To compare the deleterious effects caused by the D134S substitution in the context of CalB double mutant T138G-V190A, we analyzed conformational dynamics of the protein structure before and after aspartic acid substitution for serine. Normal Mode Analysis (NMA) was performed using the modelled structures from RosettaBackrub as input in server elNémo^76^. The elastic networks provided by NMA resulted in ten lowest-frequency normal modes (*i.e*. models). The best models were manually selected considering the best frequencies and collective scores provided by the server using default parameters.

## Supplementary information

Supporting Information
