# Supplementary material for: Dissecting the evolvability landscape of the CalB active site toward aromatic substrates
Source: Sci Rep. 2019 Oct 30;9:15588. doi: 10.1038/s41598-019-51940-0 (PMC6821916; doi:10.1038/s41598-019-51940-0)
Supplement: Supplementary file 1 — Supporting Information [file 41598_2019_51940_MOESM1_ESM.docx]

**SUPPORTING INFORMATION**

Dissecting the evolvability landscape of the CalB active site toward aromatic substrates

**Yossef López de los Santos^1^, Ying Lian Chew-Fajardo^1^, Guillaume Brault^1^ & Nicolas Doucet^1,2,*^**

From the ^1^Centre Armand-Frappier Santé Biotechnologie, ﻿Institut National de la Recherche Scientifique (INRS), Université du Québec, 531 Boulevard des Prairies, Laval, QC, Canada, H7V 1B7; ^2^PROTEO, the Québec Network for Research on Protein Function, Engineering, and Applications, 1045 Avenue de la Médecine, Université Laval, Quebec City, QC, Canada, G1V 0A6.

Running title: *Dissecting the evolvability landscape of CalB*

*To whom correspondence should be addressed: Nicolas Doucet, ^1^Centre Armand-Frappier Santé Biotechnologie, ﻿Institut National de la Recherche Scientifique (INRS), Université du Québec, 531 Boulevard des Prairies, Laval, QC, Canada, H7V 1B7; Email: nicolas.doucet@inrs.ca; Tel. (450) 687-5010, ext. 4212; Fax. (450) 686-5501.

**Keywords:** Lipase, directed evolution, natural flavors, aromas, protein engineering, enzyme catalysis, biotechnology.

__________________________________________________________________________________

**Table SI.** Increase in synthetic activity of CalB variants.

| **Evolutionary trajectory** | **Identity** | **Activity fold increase relative to WT CalB** |
| --- | --- | --- |
| **Methyl cinnamate production** | **D134S** | **2.39** |
|  | **D134T** | **2.36** |
|  | **D138N** | **3.05** |
|  | **T138A** | **3.52** |
|  | **T138S** | **4** |
|  | **T138G** | **3.17** |
|  | **T138G-D134S** | **5.21** |
|  | **T138G-V190A** | **5.56** |
|  | **T138G-I189T** | **2.35** |
| **Methyl salicylate production** | **V154L** | **2.28** |
|  | **V154L-L278A** | **3.65** |
|  | **V154L-I189S** | **2.2** |
|  | **V154L-I189T** | **2.47** |

**Table SII.** Changes in CalB active-site volume upon mutagenesis and selection.

| **Model** | **Volume (Å^3^)** | **Area (Å^2^)** | **Cavity mouth area (Å^2^)** |
| --- | --- | --- | --- |
| **WT CalB** | **531** | **418.6** | **74** |
| **V154L** | **276.5** | **300.4** | **57.2** |
| **V154L-L278A** | **384** | **335** | **102.7** |
| **T138G** | **580** | **450** | **74.6** |
| **T138G-V190A** | **519.5** | **423.8** | **97.2** |

**Note:** Volumes were calculated with the CASTp server (1).


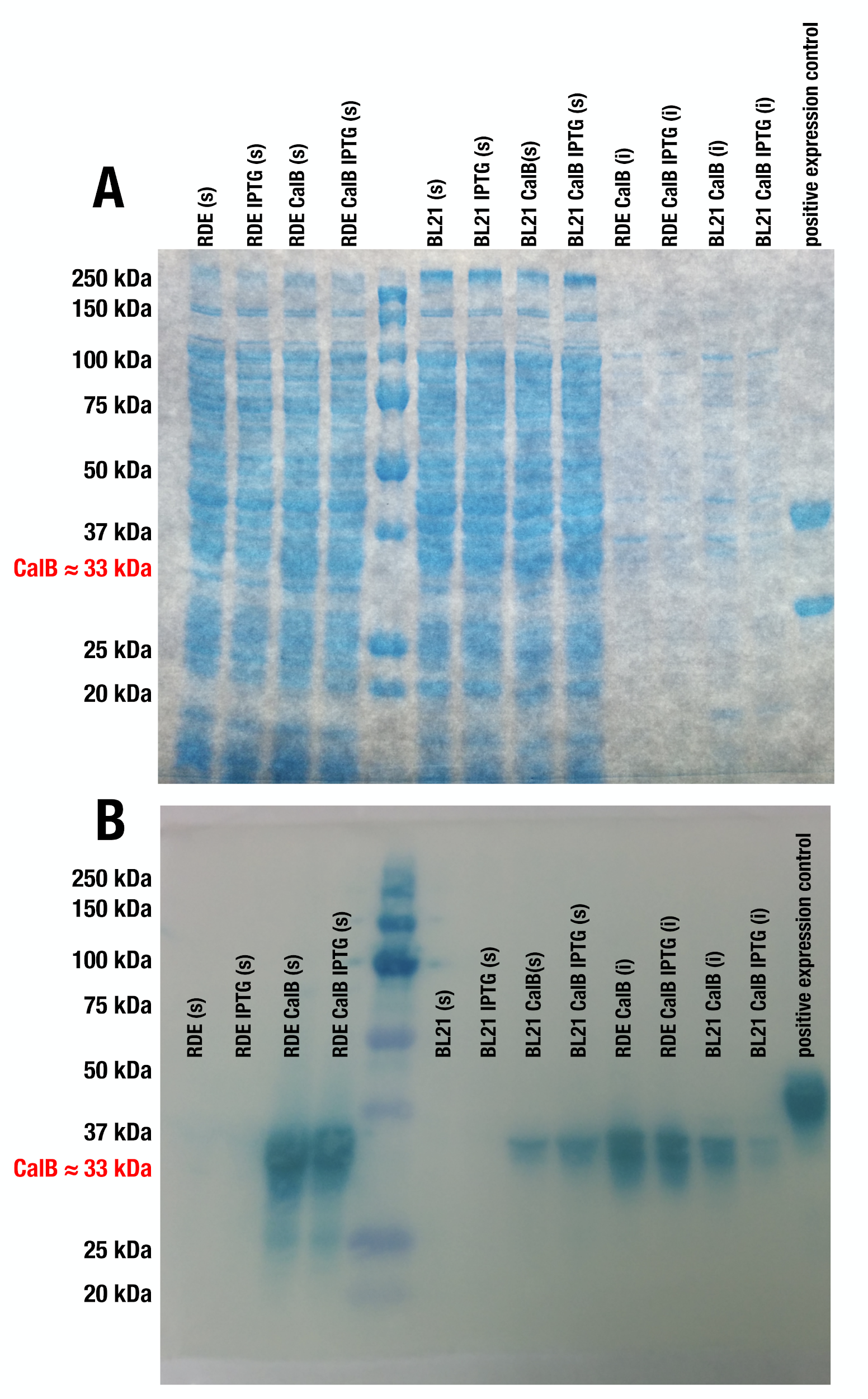


**Figure S1.** Wild-type CalB overexpression in *E. coli*. A) Comparative expression yields between *E. coli* Rosetta DE3 (RDE) and BL21 DE3 (BL21). Coomassie-stained SDS-PAGE illustrates two phenotypic expression backgrounds with and without IPTG induction for soluble (s) and insoluble (i) forms of CalB, which are respectively observed in the supernatant or expressed as inclusion bodies in cell pellets. B) Western-Blot analysis showing that the WT pET22b(+)-CalB cell system is leaky, displaying significant protein expression even in the absence of IPTG induction. See Experimental Procedures for details.

**
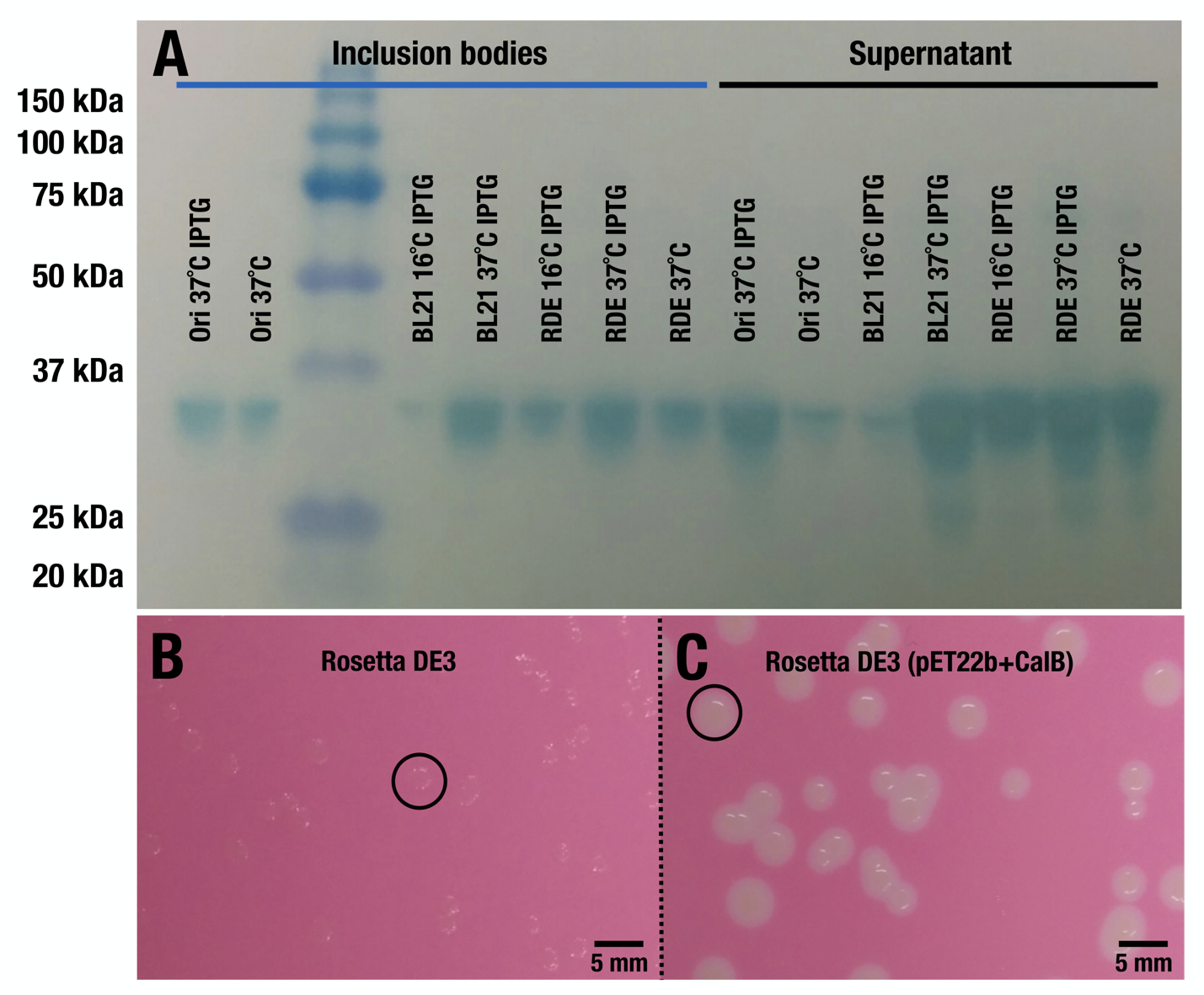
**

**Figure S2.** Combined effects of *E. coli* strain, IPTG induction, and incubation temperature on WT CalB production yields. A) Western-Blot analysis illustrates that optimal WT CalB expression is observed in the soluble extract of a Rosetta DE3 strain incubated at 16˚C. Under these conditions, CalB is not exhibiting degradation. Expression levels for soluble and insoluble extracts are shown for *E. coli* strains Origami DE3 (Ori), BL21 DE3 (BL21), and Rosetta DE3 (RDE) incubated at 16˚C and 37˚C with and without IPTG induction. B) Lipase esterification assay performed with empty strain Rosetta DE3 without expression vector or CalB expression (as negative control) on solid-state agar medium in the presence of oleic acid and decanol as substrates, in addition to rhodamine B as activity reporter (2). C) Lipase esterification assay performed with WT CalB expressed in a Rosetta DE3 background under control of expression vector pET22B+. CalB activity is revealed by the appearance of a colony halo upon enzyme overexpression, providing a suitable agar plate screening method for enzyme activity (see Experimental Procedures for details).


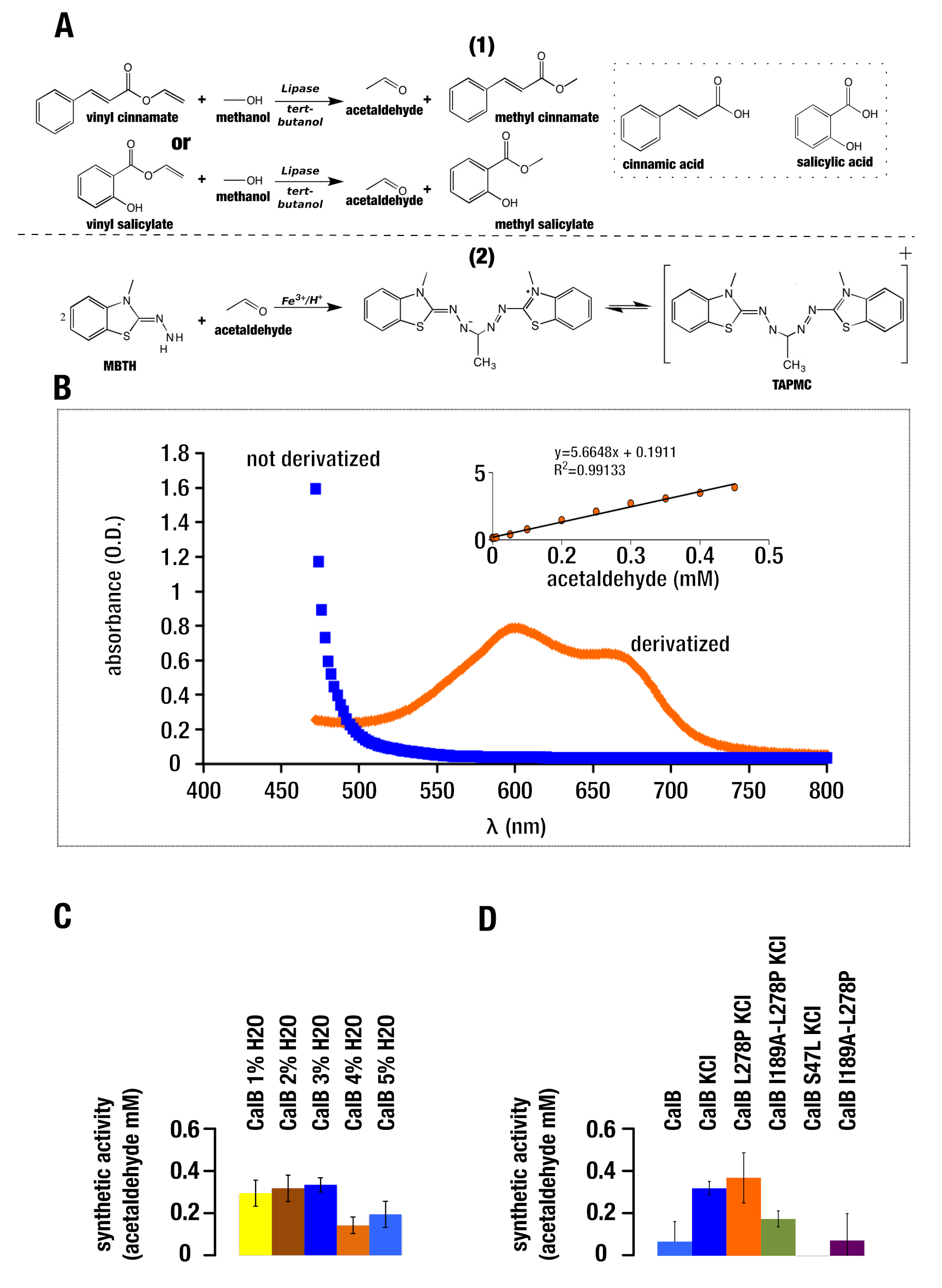


**Figure S3.** Optimization of the CalB library screening methodology for detection of methyl cinnamate and vinyl salicylate. Standardization, buffer conditions, starting template selection, and water percentage in the reaction mixture were evaluated for proper mutant selection in the enzyme engineering approach. A) Schematic representation of the reporter reaction used for screening lipase-catalyzed transesterification. The transesterification reaction is performed using the vinyl cinnamate and vinyl salicylate substrate analogs (step 1). For each enzymatic turnover with the methanol co-substrate, an acetaldehyde and flavored ester molecule (methyl cinnamate or methyl salicylate) are released in a 1:1 stoichiometric ratio. The synthetic activity is measured by a colorimetric method where 3-methyl-2-benzothialinone (MBTH) reacts with acetaldehyde to release an aldazine moiety that is converted to blue-colored tetraaza pentamethincyanine (TAPMC) (step 2) (3,4). B) UV-Visible spectral profiles of the colorimetric method employed to measure the synthetic activity of CalB in *tert*-butanol. A vinyl cinnamate or vinyl salicylate analog reacts with methanol in a transesterification reaction that releases an acetaldehyde that is further derivatized for quantifiable detection at a wavelength of 598 nm. C) A water content scan was made between 1-5 % v/v in the reaction mixture. A content of 3% H_2_O (v/v) was found optimal to measure the synthetic activity of WT CalB expressed from pET22b(+) in *E. coli* Rosetta (DE3). D) Prospective CalB template activity comparison under salt-induced activation (KCl) to promote lipase activity in *tert*-butanol (see Experimental Procedures for details). CalB variants S47L, L278P, and I189A-L278P were used as previously reported mutants showing increased synthetic activity against bulky substrates (5-7). In our experimental conditions, no significant activity improvement was observed relative to KCl-treated WT CalB.

**
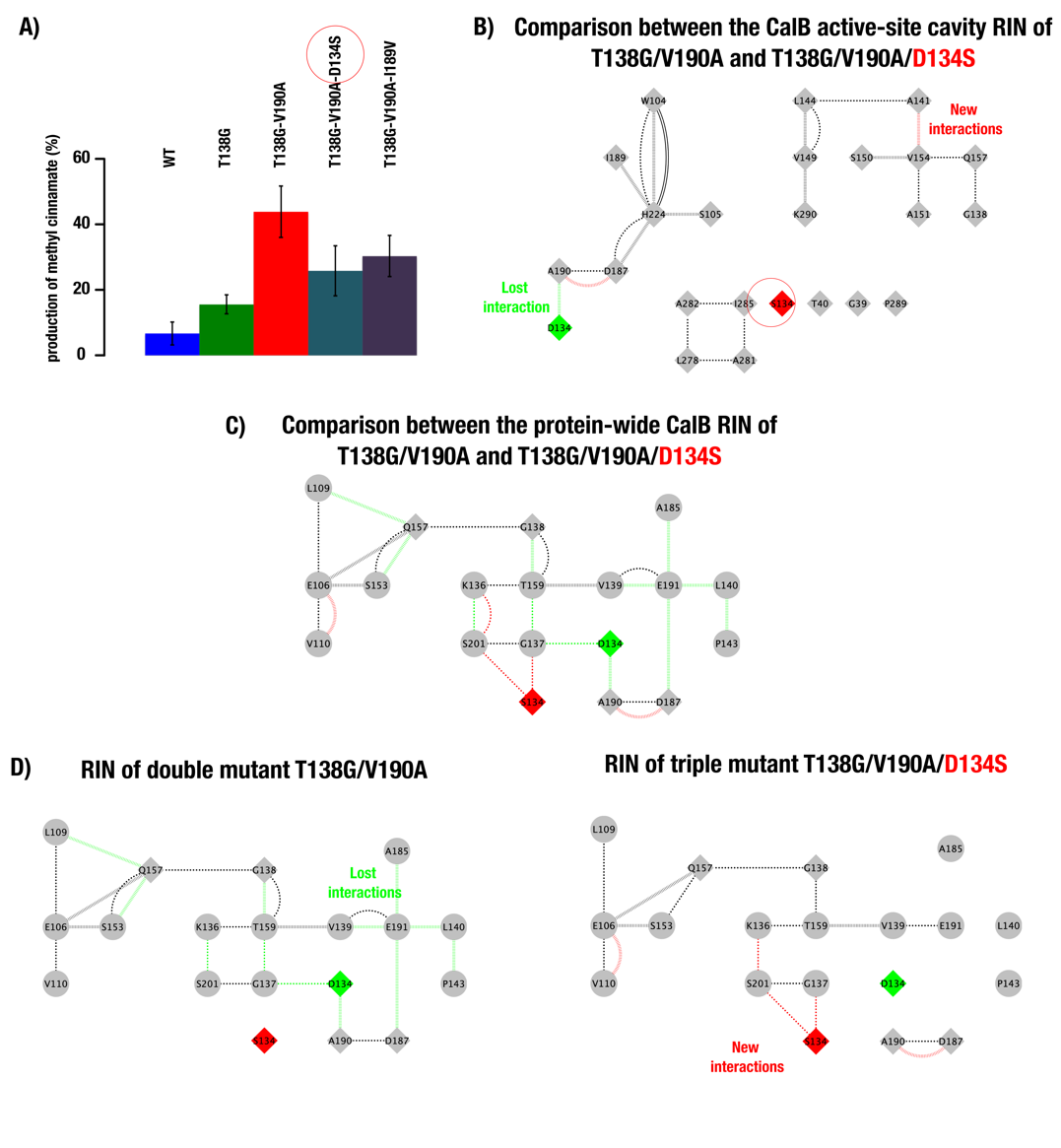
**

**Figure S4.** Residue Interaction Network (RIN) analysis of CalB upon combination of the two individually beneficial substitutions V190A and D134S. A) Deleterious impact on methyl cinnamate synthesis upon addition of the D134S substitution to double mutant T138G/V190A. The D134S substitution is highlighted by a red circle. Methyl cinnamate production by CalB variants obtained from G1, G2 and G3 library screening. Experimental conditions: 10 mg of cell extracts, 300 mM vinyl cinnamate substrate (1:3 analog:methanol ratio), 2 h incubation at 30˚C and 250 rpm. B) RIN analysis of the active-site cavity in CalB illustrates that the network of interactions is almost identical upon introduction of the D134S substitution to double mutant T138G/V190A, losing only one interaction (D134-A190, green line) while gaining two additional connections (depicted in red). WT residue D134 and mutant substitution S134 are depicted as green and red diamonds, respectively. C) Extension of the RIN analysis to the rest of the CalB enzyme, comparing the protein-wide CalB RIN of double mutant T138G/V190A with that of triple mutant T138G/V190A/D134S. Connections lost (gained) upon mutation at position 134 are presented by green (red) lines. Diamonds represent active-site cavity residues and circles represent residues outside the active-site cavity. D) Separate schemes illustrating the complete CalB RIN for double mutant T138G/V190A and triple mutant T138G/V190A/D134S. In all panels, the nature of the physical connections between the nodes (residues) is depicted by dots, vertical parallel lines, and long horizontal lines to represent hydrogen bonding, van Der Waals and ionic interactions, respectively.

**
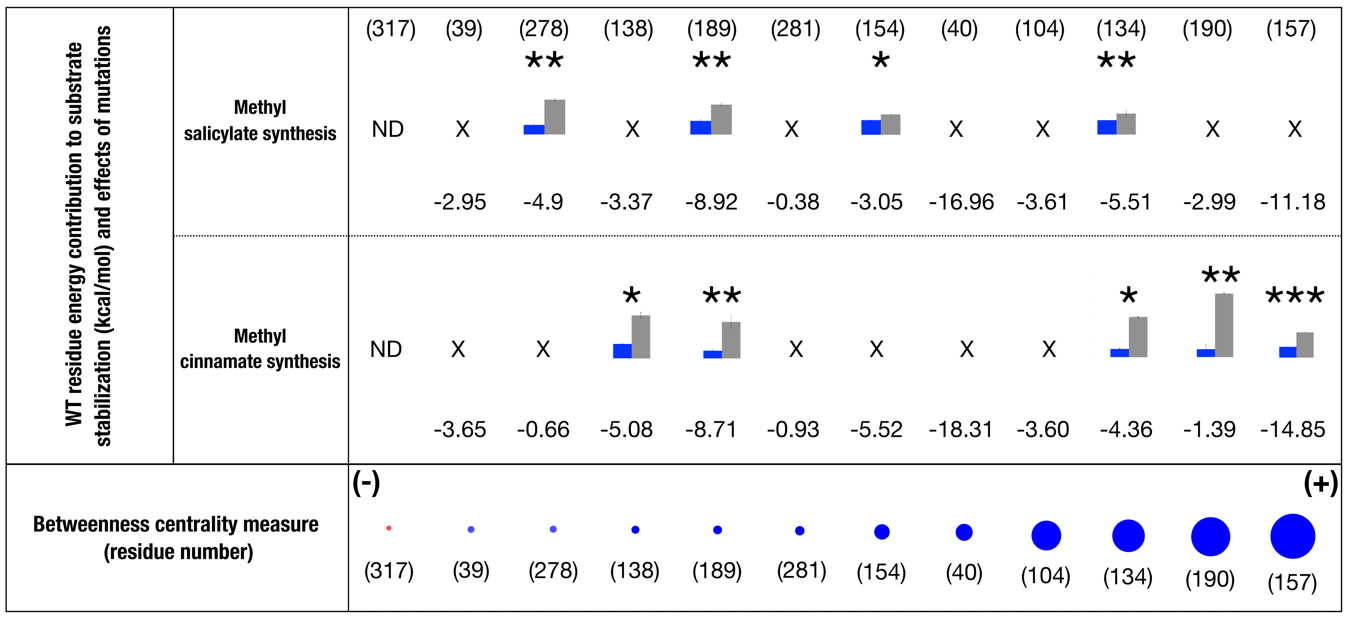
**

**Figure S5.** Comparison between centrality measure and energy contribution for residues that stabilize the vinyl salicylate and vinyl cinnamate CalB complexes. The two upper panels illustrate energy contribution per residue (negative values, in kcal.mol^-1^) and their effect after exploration of the 19 possible amino acid substitutions at selected CalB residue positions (listed on top, in parentheses). A histogram is presented when one or many mutations at these positions improves CalB activity. Detrimental effects on enzyme activity are reported by X or ND when not determined. For comparison, histograms show relative values of CalB activity for WT (blue bars) and mutants (gray bars). Asterisks delineate evolutionary generations (*i.e.* * = generation 1; ** = generation 2; *** = generation 3). The bottom panel shows values of betweenness centrality measures for positions explored in this study. Residue positions are listed in parenthesis and circles represent values of betweenness centrality measures for each WT residue. The size of the circle illustrates the importance of that residue within the entire RIN network, *i.e.* small (big) circles represent residues exhibiting low (high) values of betweenness centrality measures. For comparison, a scale is shown for betweenness centrality measures ranging from the lowest value (position 317, red circle, bottom left) to the highest value (position 157, blue circle, bottom right). Mutations that increase the catalytic activity of CalB in generations 2 and 3 (** and ***) represent substitution(s) that help improve activity only after introduction of one or two previous stabilizing mutations.

**Negative control explanation:** Residues positioned on the protein surface tend to exhibit lower connection numbers, resulting in lower chances of interrupting a shortest path. Since the active-site cavity of CalB is located near the protein surface (Figure S6), most residues that stabilize the enzyme-substrate complex have low betweenness values. Although amino acids at positions 104, 134, 157 and 190 exhibit high betweenness values (Figure S5, bottom panel), only Glu157 assembles all conditions necessary to be considered an appropriate negative control for the method used to identify hot spots that improve CalB activity. Indeed, Glu157 exhibits the highest betweenness value of the entire protein network, in addition to the second highest energetic contribution to the stabilization of the enzyme-substrate complex, just behind amino acids that play direct roles in catalysis and oxyanion hole formation (Figure S5, bottom right). It is worth mentioning that Glu157 tolerates functional substitution, but only after two rounds of stabilizing mutations. Such mutations allow functional recovery and improve CalB activity, but only after 3 generations (see evolutionary trajectory for methyl cinnamate in Figure 2 of the main manuscript).

**
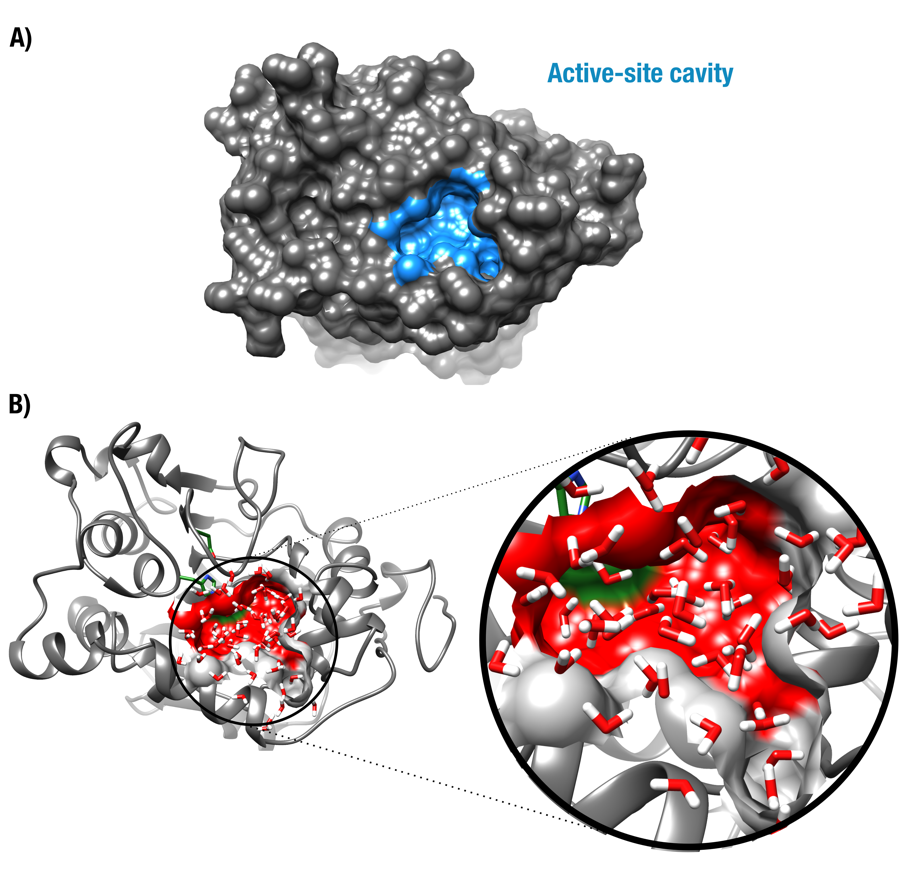
**

**Figure S6.** Residues subjected to mutagenesis within the CalB active-site cavity. A) All residues delineating the surface of the CalB active-site cavity are shown in blue. B) Solvent accessible area of the CalB cavity is illustrated by water molecules (sticks representation). The surface of catalytic residues is shown in green and the surface of residues subjected to mutagenesis in the present work is shown in red. The catalytic triad is shown as green sticks.

**REFERENCES**

1. Dundas, J., Ouyang, Z., Tseng, J., Binkowski, A., Turpaz, Y., and Liang, J. (2006) CASTp: computed atlas of surface topography of proteins with structural and topographical mapping of functionally annotated residues. *Nucleic Acids Res.* **34**, W116-W118

2. Sandoval, G., and Marty, A. (2007) Screening methods for synthetic activity of lipases. *Enzyme Microb. Technol.* **40**, 390-393

3. Zheng, J., Fu, X., Ying, X., Zhang, Y., and Wang, Z. (2014) A sensitive colorimetric high-throughput screening method for lipase synthetic activity assay. *Anal. Biochem.* **452**, 13-15

4. Zurek, G., and Karst, U. (1997) Microplate photometric determination of aldehydes in disinfectant solutions. *Anal. Chim. Acta* **351**, 247-257

5. Larsen, M. W., Zielinska, D. F., Martinelle, M., Hidalgo, A., Jensen, L. J., Bornscheuer, U. T., and Hult, K. (2010) Suppression of water as a nucleophile in *Candida antarctica* lipase B catalysis. *Chembiochem* **11**, 796-801

6. Marton, Z., Leonard, V., Syren, P. O., Bauer, C., Lamare, S., Hult, K., Tran, V., and Graber, M. (2010) Mutations in the stereospecificity pocket and at the entrance of the active site of *Candida antarctica* lipase B enhancing enzyme enantioselectivity. *J. Mol. Catal. B Enzym.* **65**, 11-17

7. Wang, F., Hou, S., Wang, Q., Wang, P., Liu, J., Yang, B., and Wang, Y. (2015) Impact of Leucine 278 residue on fatty acid length specificity of *Candida antarctica* lipase B. *Adv. Microbiol.* **5**, 493-499
